# Thermal Properties of Geopolymer Concretes with Lightweight Aggregates

**DOI:** 10.3390/ma18133150

**Published:** 2025-07-03

**Authors:** Agnieszka Przybek, Paulina Romańska, Kinga Korniejenko, Krzysztof Krajniak, Maria Hebdowska-Krupa, Michał Łach

**Affiliations:** 1 CUT Doctoral School, Cracow University of Technology, Warszawska 24, 31-155 Cracow, Poland; 2 Faculty of Material Engineering and Physics, Cracow University of Technology, Jana Pawła II 37, 31-864 Cracow, Poland; paulina.romanska@pk.edu.pl (P.R.); kinga.korniejenko@pk.edu.pl (K.K.); maria.hebdowska-krupa@pk.edu.pl (M.H.-K.); 3 Interdisciplinary Center for Circular Economy, Cracow University of Technology, Warszawska 24, 31-155 Cracow, Poland; 4 BRATA Limited Liability Company, Bałtycka 56, 76-211 Objazda, Poland; emag@brata.pl

**Keywords:** geopolymer concretes, lightweight aggregates, thermal insulation, chimney liners

## Abstract

Despite the availability of various materials for chimney applications, ongoing research seeks alternatives with improved thermal and chemical resistance. Geopolymers are a promising solution, exhibiting exceptional resistance to high temperatures, fire, and aggressive chemicals. This study investigates fly ash-based lightweight geopolymer concretes that incorporate expanded clay aggregate (E.C.A.), perlite (P), and foamed geopolymer aggregate (F.G.A.). The composites were designed to ensure a density below 1200 kg/m^3^, reducing overall weight while maintaining necessary performance. Aggregate content ranged from 60 to 75 wt.%. Physical (density, thickness, water absorption), mechanical (flexural and compressive strength), and thermal (conductivity, resistance) properties were evaluated. F.G.A. 60 achieved a 76.8% reduction in thermal conductivity (0.1708 vs. 0.7366 W/(m·K)) and a 140.4% increase in thermal resistance (0.1642 vs. 0.0683). The F.G.A./E.C.A./P 60 mixture showed the highest compressive strength (18.069 MPa), reaching 52.7% of the reference concrete’s strength, with a 32.3% lower density (1173.3 vs. 1735.0 kg/m^3^). Water absorption ranged from 4.9% (REF.) to 7.3% (F.G.A. 60). All samples, except F.G.A. 70 and F.G.A. 75, endured heating up to 800 °C. The F.G.A./E.C.A./P 60 composite demonstrated well-balanced performance: low thermal conductivity (0.2052 W/(m·K)), thermal resistance up to 1000 °C, flexural strength of 4.386 MPa, and compressive strength of 18.069 MPa. The results confirm that well-designed geopolymer lightweight concretes are suitable for chimney and flue pipe linings operating between 500 and 1000 °C and exposed to acidic condensates and aggressive chemicals. This study marks the initial phase of a broader project on geopolymer-based prefabricated chimney systems.

## 1. Introduction

Geopolymers have long been recognized as materials with high resistance to aggressive environments such as high temperatures, variable temperature cycles, moisture, and chemical agents with varying pH levels [1,2,3,4]. They also possess high mechanical properties that, along with fire resistance, make them suitable for use in chimney liners and flues [5,6,7,8,9]. One of the main limitations hindering their widespread use is their tendency to develop unsightly efflorescence on surfaces [10,11,12,13]. However, this issue is less critical when geopolymers are used in interior components, where the aesthetics are less important. Additionally, as shown in studies [14], efflorescence can be completely eliminated through heat treatment at temperatures above 600 °C. This indicates that when used in chimney liners, where high temperatures are common, these efflorescences can be removed. An essential consideration in selecting chimney construction components is the density of the materials and their insulating properties. Geopolymers can be efficiently manufactured utilizing lightweight insulating aggregates such as expanded clay. Furthermore, it is feasible to develop lightweight geopolymer aggregates that can be directly integrated into geopolymer concrete mixtures [15,16,17,18,19]. Chimney systems represent a promising application where the unique benefits of geopolymers can outweigh their known limitations.

The authors of this paper possess extensive experience in the fields of geopolymers, insulation, and fire-resistant materials, integrating both academic knowledge and industrial experience, including cooperation with a leading manufacturer and distributor of such solutions in Poland. Drawing on this practical background, this section of the introduction outlines key technical considerations and performance requirements for chimney system components based on hands-on knowledge rather than literature references. This experience provides valuable insight into the demands placed on materials that operate under highly unfavorable conditions due to the considerable temperature differences between hot exhaust gases inside the chimney and low ambient temperatures outside. Consequently, chimney blocks are mainly made of materials that withstand high temperatures and are also resistant to low temperatures (they should also have a low thermal conductivity coefficient, be lightweight, and resist temperature changes). Additionally, the specialized mortar used to join the chimney or ventilation blocks plays a crucial role, as it must also resist extreme temperature fluctuations, creating a uniform structure with the blocks. Currently, common ‘cold’ masonry mortars are used for chimney construction, where at low temperatures, the mortar layer can freeze, disrupting the chimney’s draft (e.g., causing exhaust gases to return). To ensure the proper operation of the chimney system, it is essential to prevent local cooling of exhaust gases or ventilation air, as this can cause them to descend and, consequently, backflow, which interferes with the proper functioning of boilers, fireplaces, or ventilation systems. This issue is especially evident in gravity-fed fireplaces, where exhaust gases or air can flow back into the rooms.

There is also a significant risk that applying ‘cold’ mortar to a ceramic chimney flue could lead to the cracking of the ceramic pipe, owing to the considerable disparity in thermal expansion between ceramics and mortar. Consequently, it is essential to develop innovative approaches that facilitate the construction of single-layer chimney systems utilizing specialized hollow bricks, thereby eliminating the ceramic pipe. This modification would simplify the installation process and diminish the likelihood of errors during chimney assembly. Uncontrolled exhaust gas recirculation remains one of the primary issues in existing chimney systems, primarily due to temperature differences in the chimney and the consequent inadequate draught. During low outside temperatures, such as below −10 °C, the chimney is prone to freezing, which results in the malfunction of the entire heating system. This is particularly important in the case of ‘Turbo’ chimneys, which lack technical insulation wool between the ceramic pipe and the casing. In this configuration, insulation is unfeasible since the partition serves to draw in air for combustion by condensing boilers. At low temperatures, turbulence in the chimney draft occurs, leading to freezing of the chimney structure and thereby reducing the draft or causing exhaust backflow. In colder regions, including Northern and Central-Eastern Europe, condensate within the exhaust freezes in the upper chimney section, resulting in complete blockage of the exhaust pipe and potentially causing boiler shutdowns. Hence, an effective approach is needed that allows the use of hollow bricks with a low thermal conductivity coefficient to mitigate these issues.

One of the components of mortar used in chimney systems is chloride, and increased chloride content can negatively impact structures by causing deposits or efflorescence on the surface of the masonry and trapping moisture inside it. Furthermore, the mortar currently employed contacts stainless steel inserts installed within chimneys, resulting in unfavorable passivation. In existing masonry mortar solutions for chimneys, it is crucial to wait a minimum of 36 h for the chlorides in the mortar to evaporate prior to the installation of the acid-resistant pipe. However, in practice, installation teams, driven by the desire to complete their tasks swiftly, often omit this critical waiting period and proceed directly to the installation of the acid-resistant pipe, thereby generating operational issues at a later stage, such as reduced service life. Consequently, the development of efflorescence-resistant mortars suitable for simplified, single-layer chimney systems constructed from hollow bricks is of significant importance.

This analysis of current requirements and material challenges indicates that many of these issues can be addressed with geopolymer-based solutions, which have been known for several decades [20,21,22,23,24]. Geopolymers offer a set of properties that are important for chimney applications. Their implementation in this industry can significantly reduce the carbon footprint of building infrastructure while increasing durability and resistance to aggressive exhaust environments [25,26,27,28,29]. Geopolymer concretes are innovative building materials that provide a sustainable alternative to traditional Portland cement-based concretes [30,31,32,33]. They are synthesized through the alkaline activation of aluminosilicate materials, such as fly ash, slag, or other industrial by-products, combined with alkaline activators like sodium hydroxide (NaOH) and sodium silicate [34,35,36,37]. This process results in a three-dimensional network of inorganic polymers, giving geopolymer concretes unique properties, including high compressive strength, durability, and resistance to various environmental factors [38,39,40,41,42,43]. Ongoing research is needed to develop materials tailored for this application, as well as studies under real-world conditions and the development of standards for certifying geopolymer materials in chimney technology.

In recent years, there has been a notable rise in interest in lightweight geopolymer concretes (LWGCs), which demonstrate superior thermal insulation properties relative to traditional concrete materials. Multiple studies from 2024 to 2025 suggest that by appropriately selecting raw materials and additives, it is possible to effectively reduce the thermal conductivity coefficient without significantly compromising the mechanical characteristics of the material. A crucial factor is the utilization of porous aggregates—such as expanded glass, pumice, or perlite—that, due to their entrapped air content, serve to restrict heat transfer through the structural matrix. Xu and colleagues [44] demonstrated that recycled concrete incorporating perlite exhibits thermal conductivity values ranging from 0.24 to 0.32 W/m·K while maintaining compressive strengths between 18 and 27 MPa. Similarly, Jamal et al. [45] reported a beneficial impact of pumice aggregates on the thermal conductivity of geopolymer composites, achieving thermal conductivity (λ) values approximately in the range of 0.31 to 0.37 W/m·K.

Beyond mineral composition, the microstructure of the concrete plays an essential role. Ren et al. [46] proposed a constitutive model for LWGC that considers parameters such as the water–binder ratio, aggregate content, and fiber inclusion, which significantly influence density and, consequently, thermal insulation capabilities. Molecular studies by Liu and Ju [47], employing atomistic simulations, corroborate these findings by illustrating that the thermal conductivity of geopolymers increases with a higher Si/Al molar ratio but can be effectively reduced—by up to 40% at 5% humidity—by moisture content.

Reinforcing additives are equally vital; Gültekin [48] and Chung et al. [49] have established that the incorporation of fibers such as basalt and polypropylene not only enhances mechanical resistance but also promotes microstructural cohesion, thereby limiting heat conduction pathways. Tuncer et al. [50] highlight the effectiveness of pumice aggregate coated with polyester, which permits further reduction in thermal conductivity below 0.30 W/m·K without significant detriment to strength parameters. From an application perspective, understanding the behavior of LWGCs under varying thermal conditions is also important. Girish et al. [51] investigated geopolymer materials with fly ash and slag additives under tropical conditions (30–60 °C), finding thermal conductivity in the range of 0.71 to 0.82 W/m·K, thus affirming their appropriateness for infrastructural use in warm climates. Wu and Yao [52], alternatively, studied the influence of temperature on LWGC incorporating expanded glass, demonstrating structural stability and satisfactory insulation up to 400 °C.

An emerging area of research involves the functionalization of geopolymers for energy storage applications. Tran and colleagues [53] developed a graphene-enhanced geopolymer with high thermal stability and potential for heat storage, which could be applicable in passive building systems and heat recovery technologies. Additionally, study [54] evaluates the thermal efficiency of hollow bricks integrated with phase change materials (PCMs) across various climate zones. Through numerical simulations and experimental validation, the findings indicate that PCM integration substantially reduces heat transfer through partitions, stabilizes internal temperatures, and diminishes cooling energy demands in hot climates. Geopolymer concretes with lightweight aggregates exhibit properties that can be precisely tuned via strategic material engineering, which includes the careful selection of aggregates, regulation of the Si/Al ratio and moisture content, and utilization of functional additives. Such materials represent a promising solution to the challenges associated with energy efficiency and sustainable construction.

This study presents the development and validation of an innovative geopolymer concrete incorporating lightweight foamed geopolymer aggregates, designed specifically for chimney system applications. The proposed material combines high thermal resistance and good insulating performance with significantly reduced weight, meeting the strict design requirement of a maximum 18 kg per structural element (240 mm × 240 mm × 330 mm) and 60 kg per running meter of the complete chimney system. This represents a substantial technological advancement over conventional solutions, which are typically much heavier and less suitable for lightweight or modular construction. Unlike previous research that has focused mainly on the general mechanical, chemical, or environmental performance of geopolymers, this work addresses an unstudied niche: the use of geopolymer concretes in chimney components, particularly in suspended and weight-restricted systems. No prior studies in the scientific or industry literature have proposed geopolymer mixes that simultaneously satisfy the combined requirements of low density, high thermal insulation, and fire resistance in this context. The novelty of this research lies in its application-driven approach, the use of diverse lightweight aggregates, including foamed geopolymer granules and controlled-crushed particles, and the successful experimental validation of the concept. The work supports the patented solution described in Polish patent PL220971B1 (“Hanging chimney”) and provides a basis for further research and implementation. It also aligns with global efforts to reduce CO_2_ emissions and promote sustainable alternatives to Portland cement.

## 2. Materials and Methods

### 2.1. Raw Materials for the Manufacture of Geopolymer Concretes and Foamed Geopolymer Aggregates

Class F fly ash, obtained from the combustion of hard coal at the Skawina Power Plant (CEZ Skawina S.A., Skawina, Poland), was used to produce geopolymer concrete and lightweight foamed geopolymer aggregates. This material served as the main precursor in the geopolymerization process and had quality parameters that met the highest standards for fly ash used in geopolymer technology. A detailed chemical analysis confirmed its suitability as an active binding component in the geopolymer matrix. In addition to fly ash, quartz sand from the Sand Pit in Świętochłowice (Świętochłowice, Poland) was added to the mixture as an inert mineral filler. To stabilize the porous structure of the foamed geopolymer aggregates and enhance their mechanical resistance, a hydraulic additive, Górkal 70 high-alumina cement, produced by Górka Cement Sp. z o.o. (Trzebinia, Poland), was used. Lightweight mineral aggregates, such as expanded clay from LECA (Gniew, Poland) and expanded perlite supplied by JAWAR (Glinojeck, Poland), were also incorporated to further modify the physicochemical properties of the mixtures. The chemical characteristics of the fly ash were determined using a SHIMADZU EDX-7200 X-ray fluorescence spectrometer (SHIMADZU Europa GmbH, Duisburg, Germany), with the results summarized in Table 1. Additionally, the particle size distribution was measured by laser diffraction using an Anton-Paar PSA 1190LD analyzer (Anton Paar GmbH, Graz, Austria), and the results are presented in Table 2.

### 2.2. Preparations of Foamed Geopolymer Aggregates

**Figure 1:**
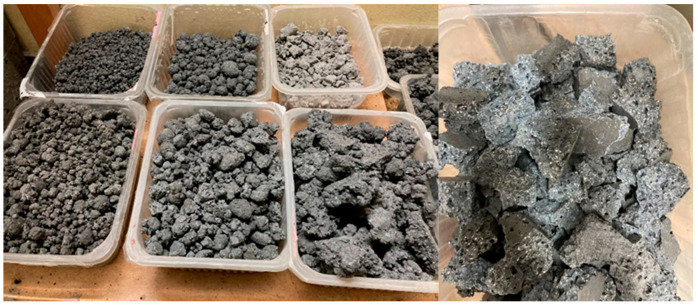
Geopolymer granules are produced by casting slabs and utilizing controlled crushing to obtain specific fractions.

The process of producing lightweight geopolymer aggregates was based on an alkaline activator in the form of a 10-molar sodium hydroxide (NaOH) solution. Technical sodium hydroxide in flake form, with a purity exceeding 99% (supplied by PCC Rokita S.A., Brzeg Dolny, Poland), was used for its preparation. The activating mixture also included an aqueous solution of sodium silicate (trade name R-145), characterized by a molar ratio of SiO_2_/Na_2_O equal to 2.5 and a density of approximately 1.45 g/cm^3^. The silicate was sourced from Zakłady Chemiczne ANSER (Wiskitki, Poland). The alkaline solution was prepared by mixing NaOH solution with sodium silicate in a weight ratio of 1:2.5, creating a stable and reactive activating liquid suitable for initiating the geopolymerization process. To impart a porous structure to the material and reduce its density, a 36% hydrogen peroxide (H_2_O_2_) solution, supplied by Grupa Azoty (Zakłady Azotowe Puławy, Pulawy, Poland), was used as a foaming agent at a proportion of 2% of the total dry mixture weight. The curing process for the foamed geopolymer mixtures was conducted under controlled thermal conditions at 75 °C. It took 24 h to reach the appropriate hardness for demolding. The material was molded into samples measuring 20 cm × 20 cm × 5 cm, then subjected to controlled crushing and screening to obtain the desired grain size. The tests utilized geopolymer aggregate with a fraction of 5–10 mm. Preliminary crushing was performed using a laboratory jaw crusher (a prototype device developed and manufactured at the Cracow University of Technology), and grain sorting was carried out on an ANALYSETTE 3 PRO laboratory sieve (MERAZET S.A., Poznań, Poland). After 28 days of curing, the samples were analyzed for their physical, mechanical, and thermal insulation properties. Special focus was given to apparent density, water absorption, compressive strength, and thermal conductivity. Photographs of the selected materials, illustrating the produced aggregates, are presented in Figure 1.

Based on previous experience [55,56,57] and the work of other authors, it was decided to produce geopolymer aggregates with the following precursor composition:

1. Base material: fly ash—38 wt.%
2. Mineral filler: quartz sand—58 wt.%
3. Stabilizer: cement (GÓRKAL 70)—2 wt.%
4. Foaming agent: 36% H_2_O_2_—2 wt.%

An alkaline solution was added to the prepared mixture to achieve a liquid-to-solid ratio of 0.4:1.

For lightweight aggregates produced with the previously described geopolymer mixture composition, it was possible to obtain materials with satisfactory mechanical and thermal insulation properties. In particular, the foamed geopolymer aggregates exhibited compressive strength exceeding 0.78 MPa, which is sufficient for many construction applications, including components of lightweight composites. Simultaneously, thermal conductivity tests were conducted on the obtained foamed geopolymer granules. Table 3 summarizes the results, showing that under optimal technological conditions, the conductivity reached 0.075 W/m × K. This low level of conductivity confirms the effectiveness of the foaming process and the stability of the porous structure, making this material particularly attractive for insulation purposes, such as chimney elements and technical casings. During the research and technological trials, it was demonstrated that the most efficient method of granulation, both technologically and economically, is controlled crushing of flat foamed geopolymer plates followed by screening to produce grains of desired sizes. Compared to traditional granulation methods in mixers with spray nozzles and rotary movement, the controlled crushing method proved to be more effective. It not only resulted in a more uniform structure but also improved rheological properties and the adhesion of aggregates within the geopolymer matrix in the final concrete. Additionally, this process is highly efficient, and any post-production waste in the form of fine fractions and dust can be recycled as an additive to subsequent batches of foamed mixtures, further enhancing the process’s efficiency and supporting circular economy principles. Due to the intended use of lightweight geopolymer aggregate in geopolymer concretes and composites, and because of the technical challenges in producing stable geopolymer foams through direct foaming and granule formation, a granulation method based on crushing already formed and hardened slabs was adopted. A comparative analysis of various forming and granulation methods demonstrated that controlled crushing not only yields high-quality final products but also enables flexible shaping of aggregate size fractions while preserving a very low bulk density, which is essential for applications in lightweight chimney systems.

### 2.3. Preparations of Geopolymer Concretes with Lightweight Aggregates

**Figure 2:**
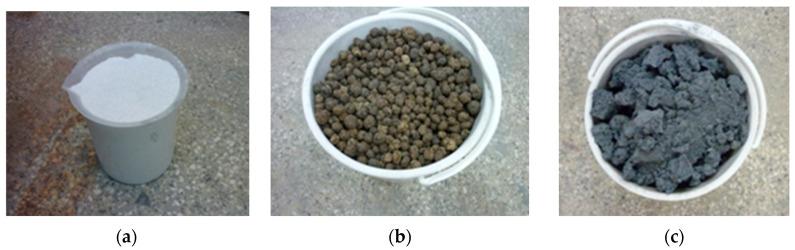
Visualization of lightweight aggregates used in the research: (**a**) perlite; (**b**) expanded clay aggregate; (**c**) foamed geopolymer aggregate.

Geopolymer concretes were produced using a standard laboratory mixer for mortars and concretes—model M/LMB-s, manufactured by GEOLAB (Warsaw, Poland). The preparation process began with the initial mixing of dry components, namely class F fly ash and quartz sand, for approximately five minutes, utilizing a mixer operating at a maximum speed of 100 rpm. After completing the dry mixing stage, a previously prepared alkaline activating solution, consisting of an aqueous solution of NaOH and sodium silicate, was slowly added to the mixture according to the specified weight ratio. The addition of the activating liquid initiated the second mixing stage, which was conducted for approximately three minutes until a uniform and homogeneous consistency of the geopolymer mass was achieved. Next, components intended to decrease the concrete’s weight and enhance its thermal insulation properties were incorporated into the mixture. These included lightweight aggregates such as foamed geopolymer aggregates and expanded clay, as well as expanded perlite serving as a lightweight filler. Specifically, lightweight geopolymer aggregates with a grain size of 5–10 mm, expanded clay aggregates measuring 10–20 mm, and expanded perlite with a grain size of 0–5 mm were introduced. Following the addition of the components, mixing was continued for an additional five minutes, this time at a reduced mixer speed of approximately 50 rpm, in order to ensure uniform distribution of the constituents and to prevent excessive damage to the porous structure of the aggregates. The resultant concrete mixture was then transferred into plastic molds and subjected to deaeration and preliminary compaction using a laboratory vibrating table. This process, which lasted approximately three minutes, was designed to eliminate excess air from the mixture and achieve the desired level of compaction. Subsequently, the samples were placed in a laboratory dryer (manufacturer: POL-EKO Perfect-Environment, Wodzisław Śląski, Poland) set at 75 °C for 24 h. These conditions were chosen to expedite the polycondensation process and stabilize the geopolymer matrix. Upon completion of annealing, the specimens were demolded and set aside for further curing and laboratory testing. All samples were prepared in the form of plates measuring 20 mm × 20 mm × 3 cm, allowing for standardized evaluation of their physical properties, thermal conductivity, and thermal resistance. Additionally, sets of samples were prepared for mechanical testing: cubic specimens measuring 10 cm × 10 cm × 10 cm for compressive strength assessment and beams measuring 16 cm × 4 cm × 4 cm for flexural strength analysis. To ensure unambiguous identification of each test series, markings were applied to facilitate classification of the composition of the mixtures. The reference sample, which did not contain any lightweight aggregates, was designated as REF. Samples containing foamed geopolymer aggregates were labeled with the symbol F.G.A. (foamed geopolymer aggregate), with the numbers 60 to 75 in the sample designations indicating the percentage by volume of the total lightweight aggregates within the concrete mixture. Furthermore, expanded clay aggregate was denoted as E.C.A. (expanded clay aggregate), whereas expanded perlite was abbreviated as P. The comprehensive composition of individual mixtures and their respective designations are provided in Table 4. Figure 2 illustrates the lightweight aggregates utilized.

### 2.4. Research Methods

#### 2.4.1. Density Measurements

The apparent density of the tested geopolymer materials was determined through a procedure involving the measurement of the sample’s mass and its geometric volume, utilizing a Lambda HFM 446 laboratory thermal properties analyzer (Netzsch, Selb, Germany). This device was adapted for the simultaneous measurement of thermal conductivity and the assessment of physical parameters of plate samples. The measurements were conducted on samples of regular plate shape (dimensions: 20 cm × 20 cm × 3 cm), which were prepared in accordance with the specified forming and seasoning procedures. Prior to measurement, the samples were carefully weighed on a precision laboratory balance (accuracy: ±0.01 g), and their volume was calculated based on geometric measurements obtained using a digital caliper (accuracy: ±0.1 mm). All measurements were performed under controlled laboratory conditions at an ambient temperature of approximately 22 ± 2 °C and relative humidity below 60%. The Lambda HFM 446 device, equipped with temperature-controlled plate measuring heads, facilitated precise determination of the thermal contact between the sample surfaces and the sensors. During the standard measurement cycle, the device automatically recorded the mass of the sample and its assigned dimensions, enabling the direct calculation of the apparent density according to Formula (1):

(1) $$\rho =\frac{m}{V}\;[\frac{kg}{m^{3}}]$$

where:

ρ—apparent density [kg/m^3^],
*m*—mass of the sample [kg],
*V*—geometric volume of the sample [m^3^].

Three independent measurements were taken for each type of sample, and the results were expressed as mean values. The obtained apparent density values were compared with the thermal conductivity measurement results to establish the correlation between the porous structure of the material and its insulating properties.

#### 2.4.2. Water Absorption Measurement

The water absorption capacity of the geopolymer concrete samples was determined in accordance with the current Polish standard PN-EN 206+A2 [58], suitably adapted to the specific characteristics of the tested materials. The procedure aimed to evaluate the samples’ ability to absorb water under fully submerged conditions. The test specimens consisted of cubes measuring 10 mm × 10 mm × 10 cm, which had been previously dried at 105 ± 5 °C until reaching a constant weight. Subsequently, the samples were cooled within a dry desiccator to an ambient temperature and then accurately weighed to determine their dry weight (*m*_0_). The samples were then immersed in a container filled with distilled water, remaining completely submerged for 48 h at room temperature (20 ± 2 °C). After this period, the specimens were extracted from the water, surface-dried using a damp cloth or paper towel, and immediately weighed to determine their water-saturated mass (*m*_1_). The saturation level was calculated based on the difference in masses, utilizing Formula (2):

(2) $$\;\;\;\;W=\frac{m_{1}-m_{0}}{m_{0}}\times 100\%\;$$

where:

*W*—absorbability [%],
*m*_0_—dry sample weight [g],
*m*_1_—weight of the sample after 48 h of soaking [g].

The test was performed in triplicate for each type of sample, and the results are presented as average values. The results obtained allowed for evaluation of the porous structure of the tested geopolymer concretes and their water absorption capacity, which is important for understanding material durability and resistance to freeze–thaw cycles.

#### 2.4.3. Flexural Strength Determination

The flexural strength test was conducted using a MATEST 3000 kN universal testing machine (Matest, Treviolo, Italy), which is suitable for precision mechanical testing of construction materials. The test procedure adhered to the requirements of EN 196-1, “Test Methods for Cement—Part 1: Determination of Strength,” particularly Section 9.1 on determining flexural strength [59]. The test specimens were rectangular, measuring 40 mm × 40 mm × 160 mm. The flexural strength (Rf) was calculated using Equation (3) for the three-point bending system:

(3) $${\;R}_{f}=\frac{1.5\times F_{f}\times l}{b^{3}}\left[\mathrm{MPa}\right]$$

where:

*R_f_*—flexural strength [MPa],
b—lateral length of the section [mm],
*F_f_*—maximum load [N],
*l*—length between supports [mm].

The test was conducted in triplicate for each type of geopolymer mixture. The resulting flexural strength values were recorded as arithmetic averages. These data serve as the foundation for assessing the mechanical resistance of geopolymer materials in the context of their potential application in structural elements and chimney systems subjected to mechanical and thermal loads.

#### 2.4.4. Compressive Strength Determination

Compressive strength tests were performed using a MATEST 3000 kN universal hydraulic press (Matest, Treviolo, Italy), adapted for mechanical testing of construction materials, including cement mortars and geopolymer concretes. The testing procedure followed the requirements of European Standard EN 196-1, “Methods for testing cement—Part 1: Determination of strength,” particularly the provisions of Section 9.2 on compressive strength [59]. Specimens measuring 100 mm × 100 mm × 100 mm were used for the tests. The compressive strength (Rc) was calculated according to Formula (4):

(4) $${\;R}_{c}=\frac{F_{c}}{A}[MPa]$$

where:

*R_c_*—compressive strength [MPa],
*A*—sample cross-sectional area [mm^2^],
*F_c_*—maximum load [N].

Three independent measurements were taken for each type of sample, and the results were expressed as mean values.

Analysis of variance (ANOVA) was conducted solely on the results of the compressive strength test, as this represents the principal mechanical property influencing the potential application of geopolymer concretes with lightweight aggregates in chimney systems. Compressive strength is a fundamental parameter for evaluating the quality and durability of construction materials, as it enables direct comparison of the efficacy of various mixtures and the assessment of the effects of additives and modifications implemented. Other tested properties, such as thermal conductivity, apparent density, and water absorption, were supplementary; their results were presented in a different format without necessitating statistical analysis.

The data obtained were crucial in further analyzing the effectiveness of geopolymer composites in terms of their suitability as lightweight, durable structural elements with insulating potential and high thermal resistance.

#### 2.4.5. Thermal Conductivity and Thermal Resistance Determination

The analysis of thermal conductivity and thermal resistance of the tested materials was conducted using a modern plate apparatus, HFM 446 Lambda from Netzsch (Selb, Germany), designed for precise measurements of insulating properties of building materials and geopolymer composites. The device operates based on the two-plate method (the so-called hot and cold plate method), in accordance with international standards such as ASTM C1784 [60], ASTM C518 [61], ISO 8301 [62], EN 12664 [63], and other established standards in thermal metrology. The HFM 446 allows testing of materials with a very broad range of thermal conductivity, from 0.007 to 2.0 W/m×K. Thanks to its high accuracy, the device achieves measurement precision of ±1–2%, a repeatability of ±0.25%, and a reproducibility of ±0.5%, ensuring reliable and consistent results. Temperature control and stabilization are maintained using Peltier modules, enabling precise replication of post-measurement conditions and rapid attainment of the desired temperature settings. Thermal conductivity was measured within the temperature range of 0 °C to 20 °C, representing typical operating conditions for insulating building materials. The samples, prepared as plates with dimensions suitable for the measuring chamber, were tested to ensure uniform temperature distribution and proper contact with the heating and cooling plates. The mass of each sample was precisely measured using a laboratory analytical balance, RADWAG PS 200/2000 R2 (Radwag, Radom, Poland), with an accuracy of 0.01 g, while the geometric dimensions (length, width, thickness) were measured using a high-precision laboratory caliper with a measurement resolution of 0.01 mm. The data collected from these measurements were used to compute the thermal conductivity coefficient (λ) and the resulting thermal resistance (R), facilitating a comprehensive evaluation of the thermal insulation properties of the tested geopolymer lightweight materials. The results served as a basis for further analysis of the material’s suitability for high-insulation applications, including, but not limited to, chimney structures with reduced dead weight and enhanced thermal resistance.

The evaluation of thermal resistance for the designed geopolymer materials was conducted in accordance with test procedures that are utilized for qualifying products intended for use in chimney systems. A crucial aspect of these tests involved the visual inspection of samples subjected to extreme heat exposure, mimicking the ignition of soot within a flue pipe. For this purpose, the samples were exposed to temperatures approximating 1000 °C, corresponding to the temperature associated with the rapid combustion of accumulated soot—a phenomenon that can occur spontaneously and unexpectedly during chimney operation. Throughout the testing process, the presence of macroscopic damage to the surface of the samples—such as cracks, delamination, or visible structural deformation—was monitored. The absence of visible damage following the test served as the basis for a positive assessment of the material’s fire resistance.

#### 2.4.6. Morphology Characterization

A high-resolution scanning electron microscope (SEM) model JEOL JSM-5510LV (JEOL Ltd., Akishima, Tokyo, Japan), coupled with an IXRF System 500 Digital Processing type energy-dispersive X-ray spectrometer (EDS), was used to analyze the morphology and microstructure of the geopolymer concretes in detail. Before imaging, the samples underwent meticulous preparation according to electron microscopy procedures to ensure the representativeness and reproducibility of the results. The preparation process included the following:

1. Preliminary cleaning of the samples to remove loose debris and dust residues that could interfere with the analysis of surface topography.
2. Gentle drying at 40 °C under controlled conditions to eliminate moisture without risking thermal degradation or chemical changes to the geopolymer structure.
3. Mounting samples on specialized preparation tables using double-sided carbon strips to ensure a stable and conductive connection to the microscope holder.

Given the non-conductive nature of the geopolymers, the samples were sputter-coated with an ultra-thin layer of gold prior to analysis using the DII-29030SCTR Smart Coater (JEOL Ltd., Peabody, MA, USA). SEM observations allowed for detailed examination of the pore structure, aggregate distribution, and geopolymer matrix, as well as the identification of microcracks and structural defects that can significantly impact the material’s mechanical and thermal properties. The combination of SEM and EDS enables a comprehensive understanding of the relationship between microstructure and macroscale performance.

#### 2.4.7. AI-Assisted Analysis

Selected sections of the manuscript, particularly Section 3 and Section 4, were linguistically enhanced and stylistically polished utilizing the artificial intelligence tool ChatGPT (OpenAI, model GPT-4, 2025 version). The AI tool was employed to support the authors in enhancing clarity, coherence, and language fluency while preserving the scientific integrity and authorship of the work.

## 3. Results

### 3.1. Physical Properties of Geopolymer Concretes with Lightweight Aggregates

**Figure 3:**
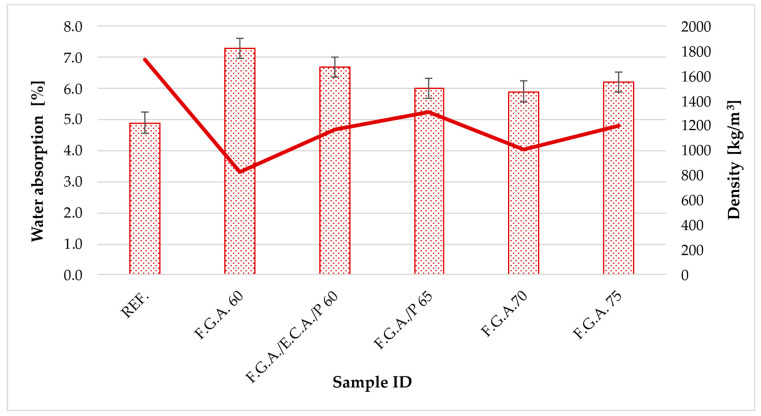
Graph of the relationship between water absorption and density.

Table 5 presents the results of testing the physical properties of geopolymer concretes with lightweight aggregates. It displays the density, water absorption, and thickness of the produced slabs. Figure 3 shows a graph illustrating the relationship between water absorption and density.

Based on the data presented in Table 5, noteworthy differences are observable in the physical properties of samples containing varying proportions of lightweight aggregates, such as foamed geopolymer, expanded clay, or perlite, in comparison to the reference sample (REF.), which serves as the benchmark and comprises only fly ash and sand in equal proportions (50% each). The reference sample was formulated without the inclusion of lightweight aggregate additives; in contrast, the samples designated as F.G.A. 60, F.G.A./E.C.A./P 60, F.G.A./P 65, F.G.A. 70, and F.G.A. 75 exhibited significantly reduced ash and sand content, favoring geopolymer foams, with some samples additionally enriched with perlite and expanded clay. Specifically, the F.G.A./E.C.A./P 60 and F.G.A./P 65 samples contained expanded clay (13.4%) and perlite (0.1%), respectively, thereby imparting a more complex material characterization. The employment of lightweight aggregates has markedly decreased the material density. The reference sample demonstrates the highest density, measuring 1735.0 kg/m^3^, attributable to its compacted structural composition devoid of porous constituents. Conversely, the incorporation of geopolymer foams led to a substantial reduction in density, reaching as low as 823.8 kg/m^3^ in the F.G.A. 60 sample. As the foam content increased, for instance, in F.G.A. 70 and F.G.A. 75, there was a gradual increase in density (to 1013.8 kg/m^3^ and 1200.0 kg/m^3^, respectively), potentially indicative of structural densification and inhomogeneous pore distribution. Samples containing foam combinations with expanded clay (F.G.A./E.C.A./P 60 at 1173.3 kg/m^3^) and perlite (F.G.A./P 65 at 1314.9 kg/m^3^) reached intermediary density values. The thickness of the samples ranged from 2.412 cm (F.G.A. 70) to 2.895 cm (F.G.A. 75), suggesting minor differences likely attributable to the forming process and the presence of air voids within the material microstructure. Analysis of water absorption revealed that the reference sample had the lowest water absorption capacity (4.9%), characteristic of materials with a dense microstructure. The introduction of lightweight aggregates resulted in increased water absorption across all other samples; however, these values remained within acceptable limits. The highest absorption rate was observed in F.G.A. 60 (7.3%), likely due to the elevated porosity of the geopolymer material in this sample. Conversely, samples F.G.A. 70 and F.G.A. 75 demonstrated lower saturation levels (5.9% and 6.2%, respectively), despite containing higher proportions of geopolymer foam, which may suggest a more closed pore structure. Samples with expanded clay (F.G.A./E.C.A./P 60 at 6.7%) and perlite (F.G.A./P 65 at 6.0%) exhibited intermediate water absorption values. The utilization of foamed geopolymer and additives such as expanded clay and perlite effectively reduces the density of the composite, with only moderate increases in water absorption. Samples containing the greatest proportion of foam (F.G.A. 70 and F.G.A. 75) present an advantageous balance between low density and acceptable water absorption, rendering them promising candidates for applications demanding lightweight yet sufficiently durable building materials.

### 3.2. Mechanical Properties of Geopolymer Concretes with Lightweight Aggregates

**Figure 4:**
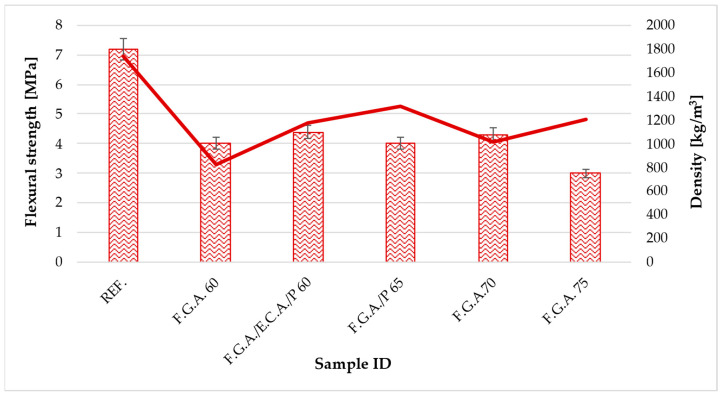
Relationship between flexural strength and density.

**Figure 5:**
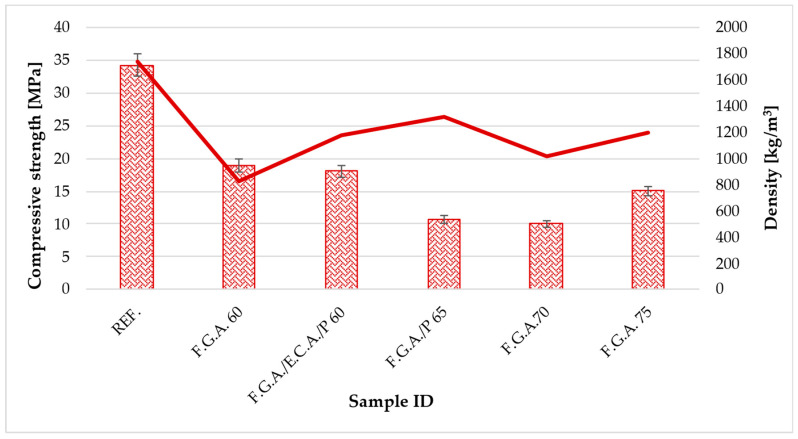
Relationship between compressive strength and density.

Table 6 shows the results of testing the mechanical properties of geopolymer concretes with lightweight aggregates. Figure 4 shows a graph of flexural strength versus density, while Figure 5 shows a graph of compressive strength versus density.

The reference sample exhibits the highest strength in both bending and compression, with values of 7.2 MPa and 34.27 MPa, respectively. This sets the standard for the other samples. All modified samples containing varying amounts and combinations of F.G.A. show a marked decrease in strength compared to the reference sample. Specifically, the flexural strength of the modified samples ranges from about 3 to 4.4 MPa, which is less than half the value of the REF. The highest flexural strength among the modified samples was achieved by the F.G.A./E.C.A./P 60 sample, reaching 4.386 MPa, while the lowest was recorded for the F.G.A. 75 sample at 2.994 MPa. Regarding compressive strength, the best results were obtained with samples F.G.A. 60 and F.G.A./E.C.A./P 60, at 18.893 MPa and 18.069 MPa, respectively—about half the strength of the REF. Notably, samples with higher proportions of F.G.A., such as F.G.A./P 65 and F.G.A. 70, show a considerably lower compressive strength of approximately 10–11 MPa, only about 30% of the REF. Conversely, F.G.A. 75 exhibits a slightly higher compressive strength of 15 MPa compared to samples 65 and 70, yet it remains significantly lower than the REF. In lightweight concrete, compressive strengths of 10–19 MPa and flexural strengths around 3–4.5 MPa are typical or acceptable, particularly for structural elements or thermal insulation with lighter loads. Since lightweight concrete generally has lower mechanical strength than regular concrete, the observed decrease is expected. The addition of F.G.A. and related combinations adversely affects the mechanical properties of the test specimens, reducing their flexural and compressive strengths relative to the REF. The most favorable mechanical performance among the modified samples was observed at lower proportions, especially in the F.G.A. 60 and F.G.A./E.C.A./P 60 samples. Increasing the percentage of F.G.A., especially to a value of 75%, results in a further decrease in flexural strength, although in this case, the compressive strength does not reach the lowest levels. Regarding industry standards for ceramic chimney linings (such as PN-EN 1457-1 for ceramic chimney inserts or guidelines for fireclay pipes [64]), the minimum compressive strength should typically be around 10 MPa. All tested mixtures, except one (F.G.A./P 65—10.703 MPa), met or exceeded this limit. Additionally, the two best mixtures (F.G.A. 60 and F.G.A./E.C.A./P 60) achieved results around 18 MPa, meaning their strength is over 52% of the reference sample value while significantly reducing density and thermal conductivity. Consequently, the mechanical results are sufficient from an operational safety and technical standards perspective for lightweight chimney linings. They also provide favorable thermal insulation properties and reduce the structure’s weight, which is essential for chimney systems installed in innovative buildings.

In order to determine whether there are statistically significant differences between the mean values for the six experimental groups—REF., F.G.A. 60, F.G.A./E.C.A./P 60, F.G.A./P 65, F.G.A. 70, and F.G.A. 75—a one-way analysis of variance (ANOVA) was conducted. This analysis revealed a clearly significant main effect (F(5.12) = 21.65; *p* = 0.0000127), indicating that at least one group differs significantly in mean value from the others. The total sum of squares (SS = 1301.94), representing the total variability in the data, was divided into a component related to differences between groups (SS_between = 1172.03, mean square MS_between = 234.41) and a component related to variability within individual groups (SS_within = 129.91, MS_within = 10.83). The calculated F-value significantly exceeds the critical value for α = 0.05 (F_crit = 3.11), allowing us to reject the null hypothesis of no differences between group means. The results indicate that variables related to F.G.A. modifications, depending on their application conditions, have a significant impact on the outcomes. This suggests that the method of preparation or composition of the sample substantially influences the system’s response. The detailed results of the analysis are shown in Table 7 and Table 8.

**Figure 6:**
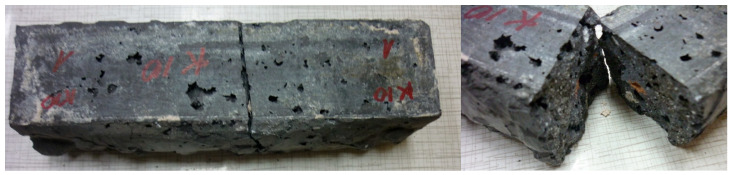
F.G.A./E.C.A./P 60 sample after the flexural strength test.

Figure 6 further illustrates an example of the specimens’ appearance following flexural strength testing. Owing to the substantial amount of aggregates incorporated, pores are observable on the surface of the specimens despite the compaction process. Visible at the fracture surface of the specimens are grains of expanded clay (orange) and lightweight aggregates of foamed geopolymer (gray), which do not markedly distinguish themselves from the geopolymer matrix.

### 3.3. Thermal Properties of Geopolymer Concretes with Lightweight Aggregates

**Figure 7:**
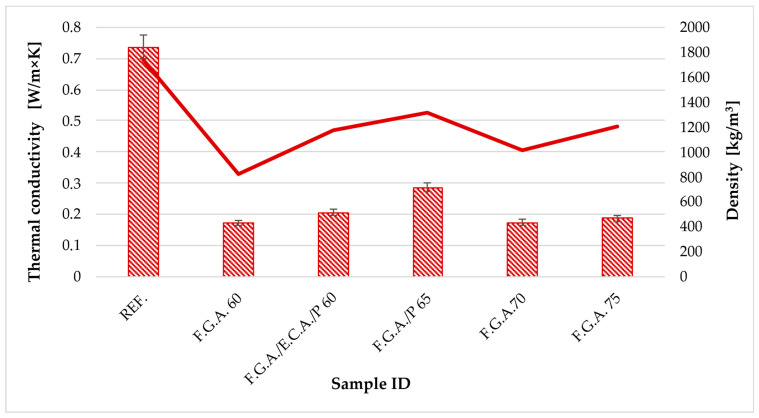
Relationship between thermal conductivity and density.

Table 9 presents the results of testing the thermal properties of the geopolymer concretes with lightweight aggregates. The table shows the results of tests of thermal conductivity, thermal resistance, and fire resistance at 800 and 1000 °C of the produced slabs. Figure 7 shows a graph of thermal conductivity versus density.

The reference sample has the highest thermal conductivity at 0.7366 W/(m·K) and the lowest thermal resistance of 0.0683 (m^2^·K)/W. This indicates that the material conducts heat much more effectively than modified samples, which is typical of denser and less insulating materials. All samples containing F.G.A. show a significantly lower thermal conductivity, ranging from about 0.17 to 0.29 W/(m·K), corresponding to a much higher thermal resistance between approximately 0.088 and 0.164 (m^2^·K)/W. The lowest thermal conductivity and the highest thermal resistance are observed in the F.G.A. 60 sample (0.1708 W/(m·K) and 0.1642 (m^2^·K)/W, respectively), highlighting its good thermal insulation properties. The F.G.A. sample also exhibits similarly advantageous insulating qualities. Regarding resistance to annealing at 800 °C and 1000 °C, the reference, F.G.A. 60, and F.G.A./E.C.A./P 60 samples show full resistance (marked as 1), indicating they maintain their properties after exposure to these high temperatures. Conversely, the F.G.A. 70 and F.G.A. 75 samples show no resistance to annealing at these temperatures (marked as 0), implying degradation or loss of mechanical properties during high-temperature exposure. Incorporating F.G.A. into the material substantially enhances its insulating properties by reducing thermal conductivity and increasing thermal resistance, which is beneficial for applications requiring effective thermal insulation. However, higher F.G.A. contents (70 and 75) may negatively impact the material’s resistance to high-temperature annealing, which should be considered during material design for such conditions. The optimal balance between insulating properties and thermal resistance appears in samples with lower F.G.A. proportions, especially F.G.A. 60 and F.G.A./E.C.A./P 60.

**Figure 8:**
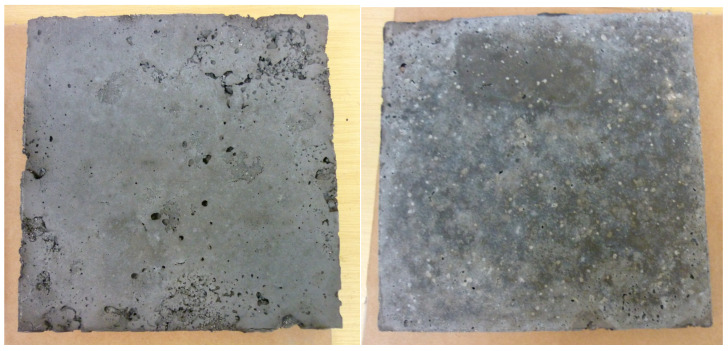
Examples of manufactured plates for thermal conductivity testing.

**Figure 9:**
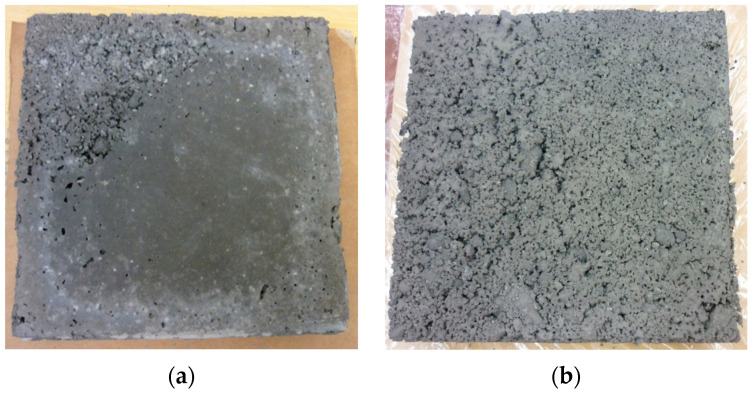
Comparison of sample surfaces: (**a**) F.G.A./P 65; (**b**) F.G.A./E.C.A./P 60 exhibiting a visibly rough surface.

Figure 8 and Figure 9 show the appearance of the samples that underwent heat conduction coefficient and high-temperature resistance tests. Not all samples had a uniform structure due to their composition. Samples with a high amount of lightweight aggregates and perlite exhibited a rough surface and numerous voids (created due to the low ratio of liquid to solid—dense consistency). To ensure consistent conditions, the samples were produced with a constant amount of liquid activator, and mixtures with more aggregates contained a higher water content. As a result, after forming, their surfaces were not smooth, as shown below in Figure 9. Additionally, it was observed that the addition of expanded perlite reduced the rheological properties and altered the mixture’s consistency. Consequently, the samples containing the perlite additive had significant surface roughness despite undergoing a compaction process.

**Figure 10:**
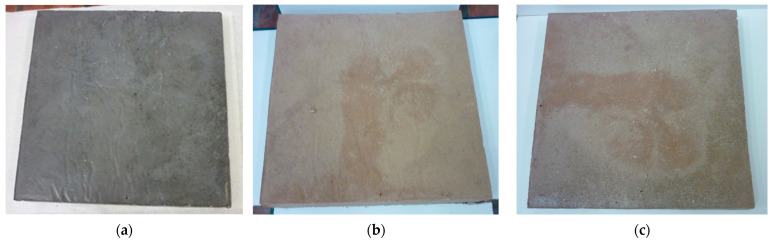
F.G.A. 75 plates: (**a**) before testing; (**b**) after exposure at 800 °C; (**c**) after exposure at 1000 °C.

**Figure 11:**
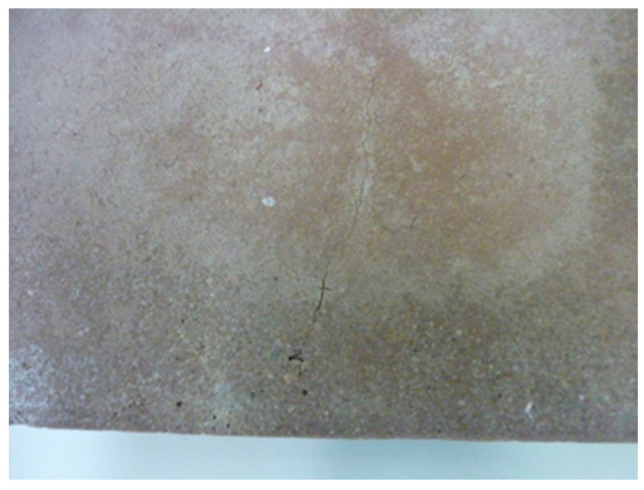
Visible surface cracking in sample F.G.A. 75 (at high magnification, a fine grid of cracks across the surface is also visible).

**Figure 12:**
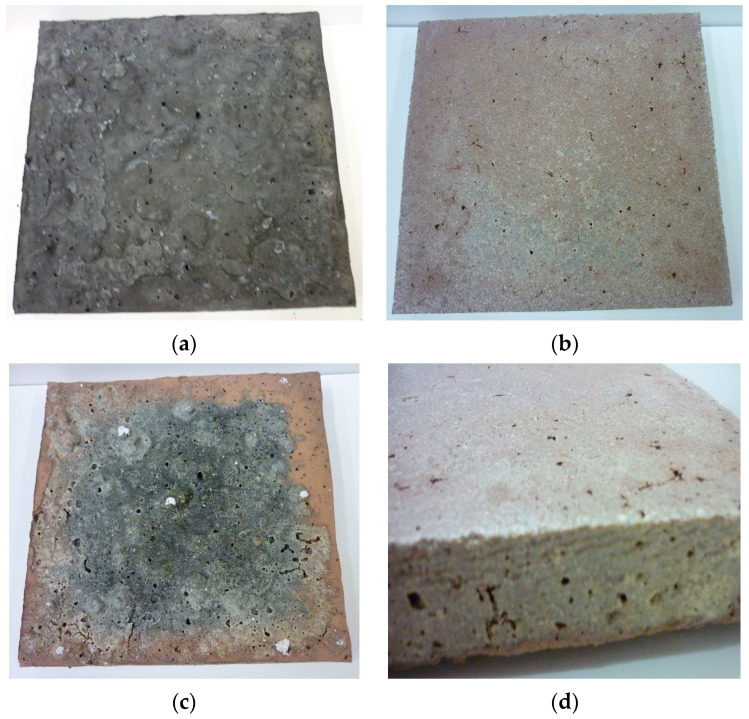
F.G.A. 70 plates: (**a**) before testing; (**b**) after exposure at 800 °C; (**c**,**d**) after exposure at 1000 °C.

High-temperature resistance tests were conducted in a laboratory oven by gradually heating the samples to 100 °C for two hours to remove moisture and prevent the rapid release of water vapor, which could cause material degradation. Then, the samples were heated to 800 °C for four hours and held at this temperature for 30 min. Some samples were transferred to a chamber in another furnace heated to 800 °C and cooled in the furnace. All samples were visually inspected for surface defects such as cracks. The remaining samples were heated to 1000 °C for 1 h, then held at that temperature for 30 min, cooled in the furnace, and inspected visually. Figure 10, Figure 11 and Figure 12 below present selected photographs showing the surface appearance of the tested samples.

### 3.4. Morphology of Geopolymer Concretes with Lightweight Aggregates

**Figure 13:**
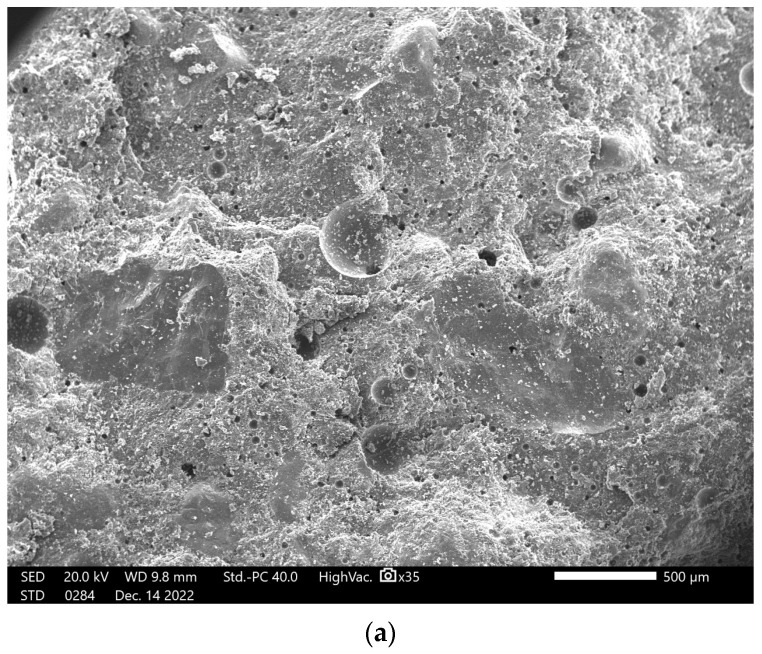

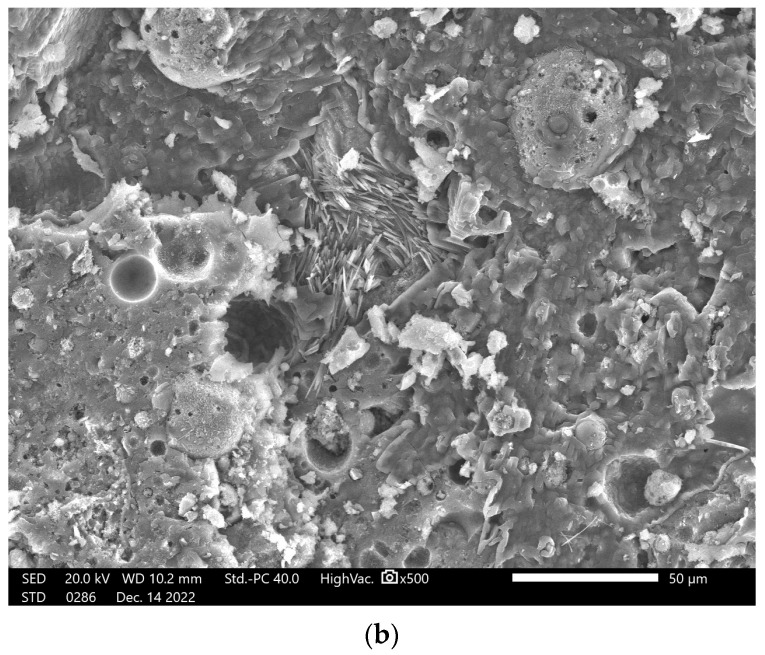
Representative SEM images of F.G.A. 60 at low (**a**) and high (**b**) magnification.

Figure 13 shows the exemplary results of morphology studies of geopolymer concretes with lightweight aggregates. The images were taken at 35× and 500× magnification.

The morphology of the tested samples of geopolymer concretes with lightweight aggregates features a porous structure with varied pore distribution and uniform dispersion of aggregate within the geopolymer matrix. Microscopic observations reveal that using lightweight aggregates creates numerous pores of different sizes, which decrease the density of the material and improve its insulating properties. In the geopolymer matrix, smooth, well-bonded phases are visible, indicating strong adhesion between components. The lightweight aggregate is evenly distributed, enabling efficient load transfer and reducing the risk of local stress concentrations. The overall morphology of geopolymer concretes with lightweight aggregates suggests a favorable structure that combines low density with acceptable strength, making them promising materials for applications requiring thermal insulation while maintaining adequate mechanical performance.

### 3.5. Assessment of Functional Properties for Geopolymer Concretes with Lightweight Aggregates

**Figure 14:**
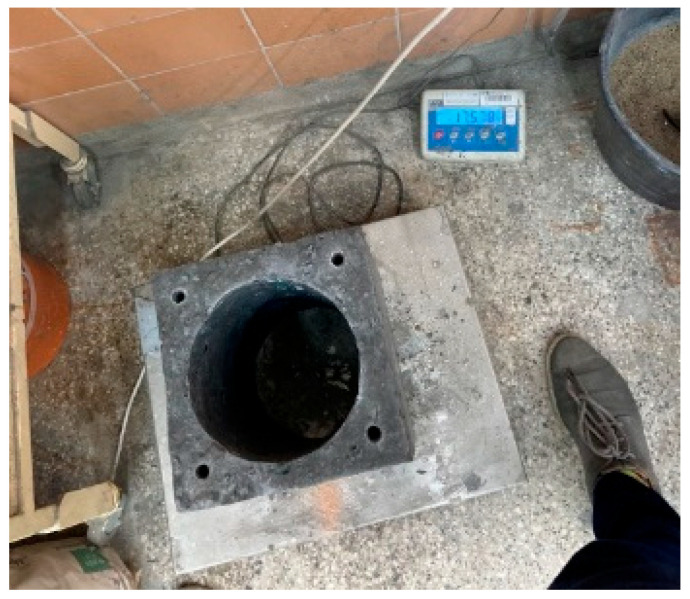
Photograph confirming the achievement of key objectives related to the purpose of the developed and tested material. The average weight of the hollow block was 17.5 kg. In no case for dimensions 240 × 240 × 330 cm did the weight exceed 18 kg, which was a basic assumption due to the need to suspend the flue on the ceiling.

**Figure 15:**
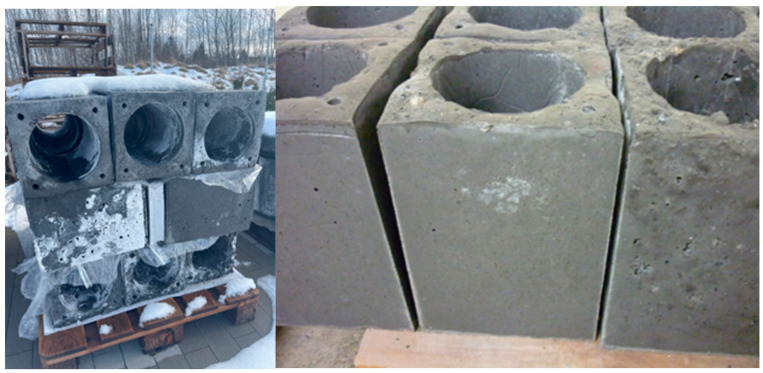
Chimney blocks made of geopolymer concrete with lightweight geopolymer aggregate—a test batch made to confirm/test under near-real conditions.

One of the key objectives of the project was to develop a construction material that would enable the building of a chimney system weighing no more than 60 kg per meter of system length. To meet this requirement, prototype chimney blocks were produced in specially prepared molds and then carefully weighed. The chimney block, made with the new geopolymer mix enriched with geopolymer and mineral aggregates, along with other filling additives, measured 240 mm × 240 mm × 330 mm and weighed less than 18 kg (Figure 14). This demonstrates that using this innovative mix results in a lightweight yet functional structural element that meets the weight criteria. Visualizations and test results of one of the completed prototypes are shown in Figure 14 and Figure 15, highlighting the quality of workmanship and confirming the weight specifications. Such a lightweight design is crucial for improving installation ease and reducing structural loads throughout the chimney system.

**Figure 16:**
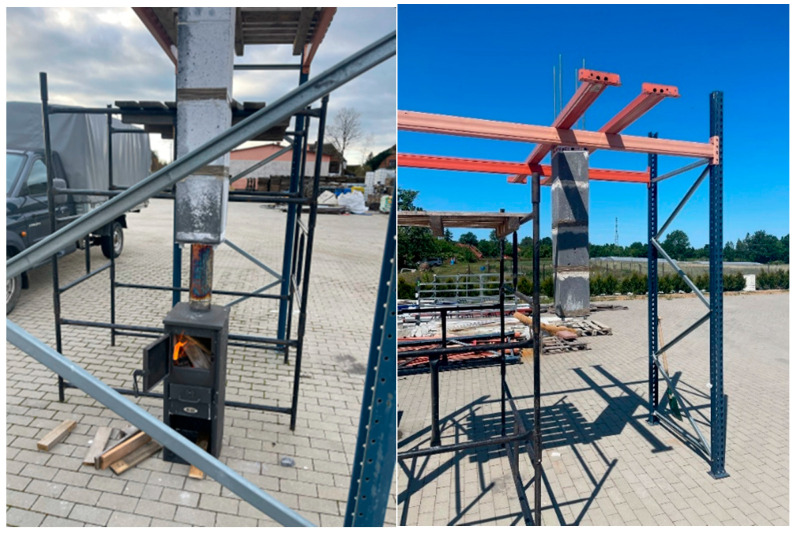
Visualization of preliminary tests of suspended flue pipes made of geopolymer concrete with geopolymer lightweight aggregate.

Figure 16 visualizes tests conducted under near-real conditions to assess the behavior of the developed geopolymer materials when exposed to different heat sources. Various heating systems were employed during the experiments, enabling a thorough analysis of the materials’ resistance to high temperatures and diverse operating environments. The accompanying photograph (Figure 15) clearly shows efflorescence on the outer surface of the geopolymer samples. This phenomenon is especially notable because the tests occurred in winter, characterized by very high humidity, which promotes condensation and the formation of efflorescence. It is important to note that the tested samples were not impregnated, which could influence the extent of the observed changes. Generally, efflorescence is common in mineral materials exposed to moisture and low temperatures, particularly without surface protection. Further research and the application of suitable impregnation methods could greatly enhance the durability and aesthetic appearance of these materials in real-world applications.

## 4. Discussion

The analysis of the aforementioned results reveals a definitive influence of the type and proportion of aggregates on the physical and mechanical properties of the geopolymer concretes. Compared to the reference material, which consists of fly ash and sand in equal parts, all compositions with the addition of foamed geopolymer and mineral aggregates (expanded clay aggregate, perlite) showed a significant reduction in density. This reduction is beneficial for decreasing the weight of the structure, especially in applications such as chimney systems, where a mass not exceeding 60 kg per meter in length is required.

The reference sample had the highest density—1735 kg/m^3^—due to its compact, low-porosity structure. The addition of lightweight geopolymer aggregates reduced the density to as low as 824 kg/m^3^ (F.G.A. 60), confirming the effectiveness of this approach. The increase in density in samples with higher amounts of foamed geopolymer (F.G.A. 70 and F.G.A. 75) may result from structural densification and a more uneven distribution of pores, a common phenomenon with a higher proportion of fillers of different sizes and types [65,66,67]. Incorporating expanded clay aggregate and perlite in the F.G.A./E.C.A./P 60 and F.G.A./P 65 samples gave the materials a more complex nature and indirectly influenced density, positioning them between the reference material and the most foamed mixtures. The measured thicknesses, ranging from approximately 2.4 to 2.9 cm, confirm a stable molding process. Variations in dimensions might be due to air pores, which also affect the insulating properties of the materials. Among the tested formulations, F.G.A. 60 demonstrates particular consistency with current research trends focused on the development of lightweight insulation materials. According to recent studies, reducing the density to approximately 800–1300 kg/m^3^ facilitates a decrease in thermal conductivity without significantly compromising mechanical performance, particularly when using pore-forming techniques and natural raw materials [68,69].

Water absorption is an important indicator of the durability and resistance of building materials. The reference sample exhibited the lowest water absorption at 4.9%, characteristic of dense, low-porosity materials. The use of lightweight aggregates increased water uptake, but the values remain within acceptable levels, which is crucial for long-term durability and weather resistance [70,71,72]. The highest water absorption of 7.3% was observed in F.G.A. 60, where the proportion of geopolymer foams was the greatest. Conversely, samples with higher densities (F.G.A. 70 and F.G.A. 75) showed lower water uptake, likely due to improved pore closure and reduced capillary action. Water absorption levels between 5.9% and 7.3%, slightly above the reference value of 4.9%, are typical results of increased porosity. In ceramic materials, high porosity decreases thermal conductivity but leads to increased water absorption and decreased strength [73,74].

In terms of mechanical strength, the results showed a decrease in both flexural and compressive strengths as the proportion of lightweight aggregates in the mix increased. The reference samples had the highest strength values, which is typical for denser materials without porosity. Nevertheless, the results obtained for concretes with lightweight aggregates, although lower, are within the acceptable range for lightweight concretes used in construction, especially where weight reduction and improved insulating properties are a priority [75,76,77]. The samples analyzed exhibited flexural strength values ranging from 2.99 to 4.39 MPa and compressive strength values ranging from 10.05 to 18.89 MPa, which are typical for porous ceramics with medium porosity. The literature reports that related materials with porosity levels between 50% and 75% exhibit strength values comparable to those obtained in this study. In Lin et al.’s study, porous 3YSZ ceramics with a porosity of 55.7% achieved a compressive strength of approximately 24 MPa [78]. In turn, Chung et al. reported that hierarchically porous SiC, produced by direct emulsion printing, with a porosity of 74%, achieves flexural strength values of 3.3 MPa [79]. Lu et al., on the other hand, reported significantly higher mechanical properties for porous silicon carbide ceramics with a considerably lower porosity of 43%. The reported values include a flexural strength of 187 MPa, a compressive strength of 215 MPa, a fracture toughness of 3.4 MPa·m^1/2^, and an energy absorption capacity of 19.2 MJ·m^−3^ [80].

In terms of thermal conductivity, the use of lightweight aggregates significantly reduced the value of this parameter, indicating an improvement in the thermal insulation properties of the materials [19,81,82]. The samples with a higher proportion of geopolymer foams exhibited the lowest thermal conductivity and the highest thermal resistance, which is especially beneficial for chimney system applications where it is crucial to prevent heat loss and reduce condensation. However, the lower thermal resistance of the samples with the highest proportion of lightweight aggregates (F.G.A. 70 and F.G.A. 75) during annealing tests suggests possible limitations for applications that require stability at very high temperatures. The thermal conductivity λ (0.1708–0.2851 W/m·K) classifies these materials among those with very good insulation properties. These values align with current research findings on porous ceramics, which exhibit thermal conductivities ranging from 0.088 to 0.193 W/m·K at high porosity, while lower values can be achieved in advanced systems such as aluminosilicate aerogels (λ ≈ 0.031 W/m·K at 1100 °C) and SiC/HfC composites (λ = 0.052 W/m·K) [83,84,85].

Morphological observations have confirmed that the use of lightweight aggregates creates a more porous structure with a varied distribution of pores, which influences the physical and mechanical properties of the material [52,86,87]. High porosity enhances thermal insulation and decreases density but also increases water absorption and reduces strength. The absence of impregnation during tests conducted under high humidity and low temperatures resulted in surface efflorescence, highlighting the need for further research on surface protection and the improvement of geopolymer material durability.

The results conclusively demonstrated that the developed geopolymer material exhibits exceptionally high resistance to extreme thermal conditions, surpassing previously employed solutions based on expanded clay concrete and ceramic building materials. Conventional chimney materials, such as expanded clay concrete, although relatively lightweight and easy to handle, are susceptible to destruction under sudden temperature fluctuations that, in practice, necessitate the replacement of entire chimney sections. The upper segments of chimneys are particularly vulnerable, where soot combustion activity is most intense. In such scenarios, structural damage frequently occurs, leading to time-consuming and costly repairs that involve dismantling roof or floor components. Ceramic bricks, while resistant to high temperatures in dry conditions, can rapidly degrade in humid environments due to the influence of acidic condensate present in flue gases, thus limiting their long-term durability. In view of the aforementioned limitations, the geopolymer compound under development, intended for use in both chimney blocks and bonding mortars, constitutes a significant technological advancement. Owing to their excellent heat resistance and stability in humid and chemically aggressive environments, these materials not only guarantee safety under soot ignition conditions but also enhance the service life of the entire chimney system, thereby reducing the risk of failure and the necessity for repair interventions.

The results confirm that lightweight geopolymer and mineral aggregates provide a promising path to creating concretes with lower weight and better insulating properties, although they involve a trade-off between strength and environmental resistance. Optimizing formulation and manufacturing processes can contribute to materials that meet the requirements of modern lightweight and energy-efficient construction.

Analysis of the optimal solution:

- F.G.A. 60 exhibits the lowest density and thermal conductivity, good strength, thermal resistance, and superior insulating properties. However, it also demonstrates the highest water uptake.
- F.G.A./E.C.A./P 60 offers a reasonable compromise—it remains lightweight, provides good thermal insulation, has reasonable absorption, and its mechanical strength is only slightly lower than F.G.A. 60 while preserving thermal resistance.
- F.G.A./P 65 has a higher density, worse thermal conductivity, and lower compressive strength than F.G.A. 60 and F.G.A./E.C.A./P 60.
- REF. has the best mechanical strength and temperature resistance, but it is heavy and insulates heat very poorly.
- F.G.A. 70 and 75 offer good thermal insulation but fail tests at 800 and 1000 °C, which disqualifies them for chimney applications.

The best material in terms of balanced properties for lightweight, thermal insulating chimney system applications is F.G.A./E.C.A./P 60. It meets the mechanical requirements, provides good insulation, acceptable absorption, low weight, and full resistance to temperatures up to 1000 °C. If the goal is maximum lightness and insulation with minimal weight, and an absorbability of 7.3% is acceptable, F.G.A. 60 would also be an excellent choice. Table 10 lists all the parameters of the sample with the best properties.

Manufacturers of such materials indicate that the main needs of customers revolve around several key aspects. First and foremost, lightweight structures that facilitate easier and faster installation are expected, which is important for both contractors and end users. Another significant feature is that the chimney system can be freely placed anywhere in the building, providing greater design flexibility and allowing better adaptation to individual architectural conditions. An additional crucial requirement is the reduced absorbability of the material, minimizing the risk of unsightly efflorescence caused by moisture and weathering. High acid resistance is another essential factor, protecting the chimney from chemical degradation, which can significantly shorten its lifespan. Equally important is the material’s ability to withstand cyclic cleaning, essential for maintaining safety and efficiency during daily operation. A notable trend is the use of modern panoramic four-pane inserts, which enhance chimney system efficiency and improve thermal insulation. In turn, good insulation properties of the chimney are vital for reducing heat loss and preventing condensation, thus improving the building’s energy efficiency. The innovative chimney system outlined in the article, produced using modern geopolymer mixtures and lightweight aggregates, addresses all these user needs. Furthermore, it complies with stringent standards for chimney systems while ensuring high quality, durability, and safety. All these factors contribute to the increasing interest and demand for such building materials, including advanced chimney systems. Rising customer awareness and their increasing demands for safety, airtightness, resistance to cleaning, and aesthetics make innovative technological solutions more attractive on the market. These systems eliminate issues like brick erosion, have high weather resistance, and maintain an aesthetic appearance over many years. Additionally, the use of such advanced materials and designs supports sustainable development principles, which are becoming more important for investors and end users. As a result, not only is the demand for modern chimney systems growing, but expectations for their properties are also rising, often surpassing current legislative requirements. This creates a need for further research and technological advancements to meet both current and future challenges in the construction market.

## 5. Conclusions

Based on the study, the following conclusions can be drawn:

- The use of lightweight porous aggregates (foamed geopolymer, expanded clay, and perlite) enabled a reduction in the density of geopolymer composites by up to 52.5% compared to the reference material (from 1735 to 823.8 kg/m^3^), which is especially beneficial for designing lightweight chimney systems and prefabricated installation elements.
- The introduction of lightweight aggregates increased water absorption to a maximum of 7.3%, but all samples stayed within a safe range, ensuring the material’s durability when exposed to moisture and acidic condensates.
- The mechanical strength of materials with added lightweight aggregates was lower than that of the reference concrete (REF.: 34.27 MPa), but some compositions, such as F.G.A./E.C.A./P 60, reached over 18 MPa, which is enough for structural and assembly applications in chimney construction.
- Thanks to a significant reduction in thermal conductivity—to 0.1708 W/(m·K)—the composites demonstrated an improvement in insulating properties of over 76% compared to the base material, which helps lower heat loss and boosts the energy efficiency of the system.
- Resistance to high temperatures (800–1000 °C) showed that most mixtures maintain dimensional and structural stability, although further optimization of the proportion of lightweight aggregates may further improve thermal resistance and prevent degradation during long-term operating cycles.
- The increased porosity of the composites improves their thermal insulation, but it reduces mechanical strength, requiring further research into microstructural enhancements and possible impregnation methods.

Research confirms that lightweight geopolymer concretes with the addition of porous aggregates—such as foamed geopolymer, expanded clay aggregate, or perlite—can effectively meet the strict requirements for materials used in chimney systems. The developed composites combine low specific weight (below 1200 kg/m^3^) with sufficient mechanical strength (≥15 MPa), good chemical resistance, and significantly reduced thermal conductivity (up to 75% less compared to the reference material), making them a practical replacement for traditional ceramic and concrete materials. The use of porous aggregates has enabled a favorable balance between thermal insulation, weight, and strength. This configuration is especially important for prefabricated suspended chimneys, where the low weight of the element directly influences ease of transport, installation, and the structural loads on the building. The developed material meets key standards for durability and chemical and thermal resistance and shows potential for further improvement through modifications of the mixture composition, selection of aggregate fractions, and possible surface impregnation. The research results open the door for wider use of geopolymer technology in chimney engineering, heating systems, and other sectors that require materials with high durability and low weight. This also aligns with sustainable construction practices—using fly ash and waste materials as secondary raw materials adds an ecological benefit to this technology.

## Figures and Tables

**Table 1:** Fly ash—oxide analysis.

Precursor		Oxide Composition (wt.%)					
	SiO_2_	Al_2_O_3_	Fe_2_O_3_	CaO	K_2_O	TiO_2_	SO_3_
**Fly ash**	58.41	30.35	3.83	2.19	2.04	0.85	0.53

**Table 2:** Fly ash—particle size distribution.

Material	D_10_ [μm]	D_50_ [μm]	D_90_ [μm]	Average Value [μm]	Standard Deviation [μm]
**Fly ash**	2.44	12.86	32.23	16.23	0.04

**Table 3:** Results of the thermal conductivity coefficient test for foamed geopolymer intended for lightweight geopolymer aggregates.

Setpoint Temperatures		Measured Temperatures		Thermal Conductivity	Thermal Resistance
Mean	Delta	Mean	Delta		
[°C]	[K]	[°C]	[K]	[W/(m × K)]	[(m^2^ × K)/W]
10.0	20.0	10.0	20.0	0.07596	0.3380

**Table 4:** Sample designations and corresponding material compositions.

Sample ID	Fly Ash		Sand		Lightweight Aggregates		
	[g]	[%]	[g]	[%]	Name of Additive	[g]	[%]
REF.	3000	50	3000	50	—	0	0
F.G.A. 60	2000	19.05	2000	19.05	foamed geopolymer aggregate	6500	61.9
F.G.A./E.C.A./P 60	2000	17.90	2000	17.90	foamed geopolymer aggregate	5666	50.7
					expanded clay aggregate	1500	13.4
					perlite	1 L	0.1
F.G.A./P 65	2000	17.70	2000	17.70	foamed geopolymer aggregate	7256	64.5
					perlite	1 L	0.1
F.G.A. 70	2000	15.65	2000	15.65	foamed geopolymer aggregate	8780	68.7
F.G.A. 75	1000	8.98	1000	8.98	foamed geopolymer aggregate	8142	73.06

**Table 5:** Physical properties of the tested materials (average values).

Sample ID	Density [kg/m^3^]	Thickness [cm]	Water Absorption [%]
REF.	1735.0	2.876	4.9
F.G.A. 60	823.8	2.799	7.3
F.G.A./E.C.A./P 60	1173.3	2.501	6.7
F.G.A./P 65	1314.9	2.514	6.0
F.G.A. 70	1013.8	2.412	5.9
F.G.A. 75	1200.0	2.895	6.2

**Table 6:** Results of conducted mechanical tests for geopolymer concretes with lightweight aggregates (average values).

Sample ID	Flexural Strength [MPa]	Compressive Strength [MPa]
REF.	7.200	34.270
F.G.A. 60	4.001	18.893
F.G.A./E.C.A./P 60	4.386	18.069
F.G.A./P 65	4.015	10.703
F.G.A. 70	4.310	10.051
F.G.A. 75	2.994	15.018

**Table 7:** One-way analysis of variance.

Groups	Counter	Sum	Average	Variance
REF.	3	102.81	34.270	23.91873
F.G.A. 60	3	56.679	18.893	11.79371
F.G.A./E.C.A./P 60	3	54.207	18.069	16.25855
F.G.A./P 65	3	32.109	10.703	0.192816
F.G.A. 70	3	30.153	10.051	12.67261
F.G.A. 75	3	45.053	15.018	0.120162

**Table 8:** Analysis of variance—statistically significant model.

Source of Variance	SS	df	MS	F	Value-*p*	Test F
Between groups	1172.030356	5	234.4061	21.65195	1.27 × 10^−5^	3.105875
Within groups	129.9131507	12	10.8261			
Total	1301.943507	17				

**Table 9:** Results of conducted tests of thermal properties for geopolymer concretes with lightweight aggregates (average values).

Sample ID	Thermal Conductivity [W/(m × K)]	Thermal Resistance [(m^2^ × K)/W]	Resistance to Annealing at 800 °C [/0/1]	Resistance to Annealing at 1000 °C [/0/1]
REF.	0.7366	0.0683	1	1
F.G.A. 60	0.1708	0.1642	1	1
F.G.A./E.C.A./P 60	0.2052	0.1219	1	1
F.G.A./P 65	0.2851	0.0882	1	1
F.G.A. 70	0.1737	0.1388	0	0
F.G.A. 75	0.1869	0.1550	0	0

**Table 10:** Parameters of the composition with the best overall properties.

Properties of the Best Sample	F.G.A./E.C.A./P 60
Density [kg/m^3^]	1173.3
Thickness [cm]	2.501
Water absorption [%]	6.7
Flexural strength [MPa]	4.386
Compressive strength [MPa]	18.069
Thermal conductivity [W/(m × K)]	0.2052
Thermal resistance [(m^2^ × K)/W]	0.1219
Resistance to annealing at 800 °C [/0/1]	1
Resistance to annealing at 1000 °C [/0/1]	1

## Data Availability

The original contributions presented in this study are included in the article. Further inquiries can be directed to the corresponding authors.
